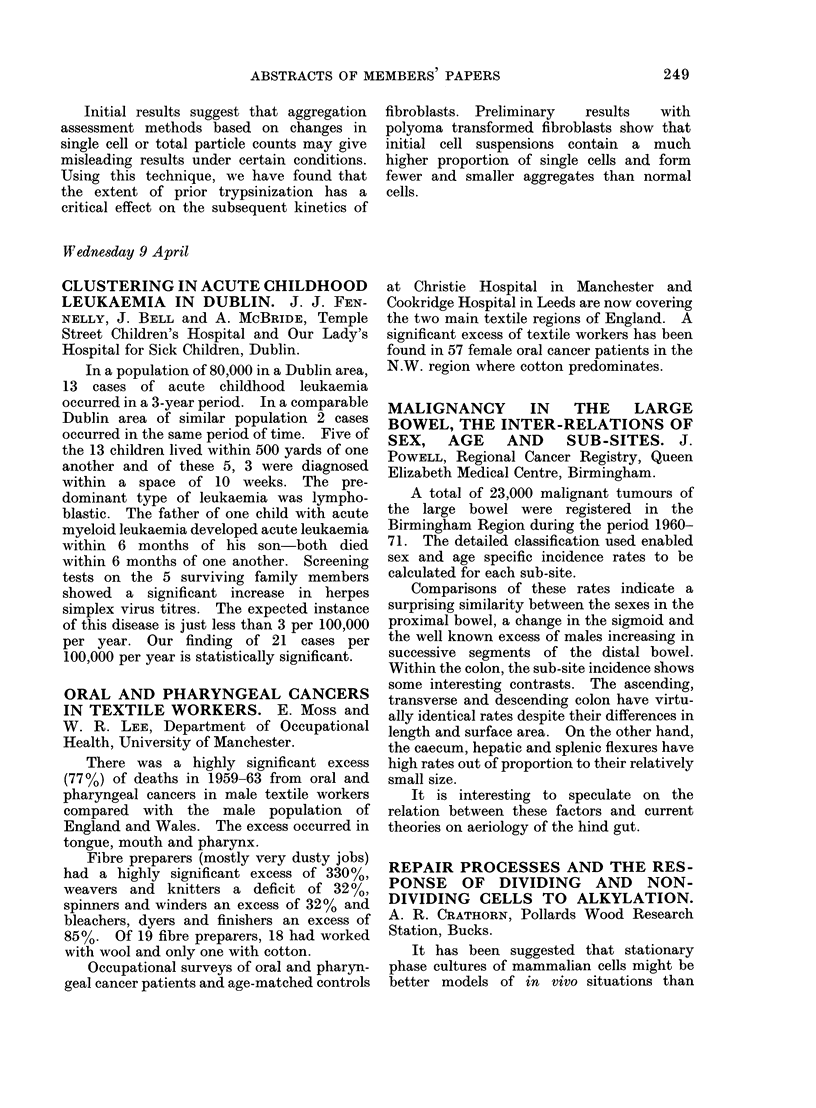# Proceedings: Malignancy in the large bowel, the inter-relations of sex, age and sub-sites.

**DOI:** 10.1038/bjc.1975.186

**Published:** 1975-08

**Authors:** J. Powell


**MALIGNANCY IN THE LARGE BOWEL, THE INTER-RELATIONS OF SEX, AGE AND SUB-SITES.** J. Powell, Regional Cancer Registry, Queen Elizabeth Medical Centre, Birmingham.

A total of 23,000 malignant tumours of the large bowel were registered in the Birmingham Region during the period 1960–71. The detailed classification used enabled sex and age specific incidence rates to be calculated for each sub-site.

Comparisons of these rates indicate a surprising similarity between the sexes in the proximal bowel, a change in the sigmoid and the well known excess of males increasing in successive segments of the distal bowel. Within the colon, the sub-site incidence shows some interesting contrasts. The ascending, transverse and descending colon have virtually identical rates despite their differences in length and surface area. On the other hand, the caecum, hepatic and splenic flexures have high rates out of proportion to their relatively small size.

It is interesting to speculate on the relation between these factors and current theories on aeriology of the hind gut.